# The impact of COVID-19 on professional development for physiotherapists in Lagos, Nigeria

**DOI:** 10.4102/sajp.v79i1.1892

**Published:** 2023-11-06

**Authors:** Atirola A. Obileye, M. Anne Chamberlain, Rory J. O’Connor

**Affiliations:** 1 Leeds Institute of Medical Education, Faculty of Medicine and Health, University of Leeds, Leeds, United Kingdom; 2 Academic Department of Rehabilitation Medicine, School of Medicine, Faculty of Medicine and Health, University of Leeds, Leeds, United Kingdom; 3 National Demonstration Centre for Rehabilitation, Leeds Teaching Hospital NHS Trust, Leeds, United Kingdom

**Keywords:** rehabilitation, physiotherapy, Nigeria, continuous professional development, COVID-19

## Abstract

**Background:**

Continuous professional development is important to maintain standards of care in the healthcare sector. However, in Lagos, Nigeria, the additional burden of COVID-19 and limited resources may provide separate challenges for physiotherapists’ continuous professional development (CPD).

**Objectives:**

To examine the availability and perceived quality of continuous professional development opportunities for physiotherapists working before and during the pandemic in Lagos, Nigeria.

**Method:**

A qualitative study was conducted with 10 conveniently sampled physiotherapists, recruited via email. Interviews took place via Zoom, and the video function was utilised. Data were collected via semi-structured interviews using a pilot tested interview, and was transcribed and analysed thematically.

**Results:**

The main method of workplace teaching pre-COVID-19 and during COVID-19 was bedside teaching (BT), which most participants received. Pre-COVID-19, the main barrier to receiving teaching was a lack of national guidelines providing specific details on CPD. During COVID-19, the main barrier was the difficulty of increased online teaching instead of teaching on real life patients. The main barriers for non-workplace CPD pre-COVID-19 were a lack of availability of learning opportunities and monetary cost of conferences. This was partially combatted by the increasing trend of online learning events during the pandemic, which increased access to non-workplace learning opportunities.

**Conclusion:**

Because of COVID-19, most CPD learning opportunities for physiotherapists in Lagos, Nigeria, were online, increasing overall accessibility. Adequate training to improve utilising online learning resources as well as specific guidelines for workplace physiotherapists CPD in Nigeria should be implemented and promoted to improve confidence and quality of care.

**Clinical implications:**

Key insight into the CPD experiences of physiotherapists currently working in Lagos, Nigeria, which can guide policies and improve clinical outcomes.

## Introduction

The Global Burden of Diseases study conducted in 2019 estimated that over 2.4 billion people worldwide are living with conditions that would benefit from rehabilitation (Cieza et al. 2020). Nigeria on the west coast of Africa has the continent’s highest population of 213 million people (World Bank 2021). Low- and middle-income countries (LMICs) such as Nigeria have a higher prevalence of disabilities because of the high incidences of trauma (motor vehicle incidents etc.,), and also on account of lack of accessible rehabilitation services such as physiotherapy (Sarobidy et al. 2020). The lack of accessibility is partly because there are only 2500 practising physiotherapists in Nigeria (0.13 per 10 000 population; World Physiotherapy 2021), limited resources as well as a lack of national guidelines for continuous professional development (CPD) in Nigeria. The few physiotherapists who have resources such as advanced equipment and additional skills from extensive CPD will often work privately and charge higher rates for their services. This makes them inaccessible to the majority of the population (Abaraogu, Edeonuh & Frantz 2016; Onyeso et al. 2022). There is limited research that has explored the quantity and impact of CPD and opportunities to benefit from CPD for physiotherapists in LMICs.

Generally, CPD is ‘the process to maintain, develop and enhance skills, knowledge and competence to improve performance at work’ (Gunn & Goding 2009). Within physiotherapy there is a broad range of activities that fall under this banner such as: bedside teaching (BT), attending conferences, networking with colleagues, and reading the professional literature. Studies have found the effects of physiotherapists engaging in and receiving CPD to be an increase in confidence and competence as well as reducing the physical disability experienced by patients (Gunn & Goding 2009; Lowe & Jackson 2008). In Nigeria, the Medical Rehabilitation Therapists Board (MRTB) regulates the clinical practice of physiotherapists and requires proof of a certain level of CPD (through a points system) to maintain a licence (Institute for Health Metrics and Evaluation [IHME] 2021). However, there is neither a detailed national standard curriculum for learning once qualified as a physiotherapist nor specific national guidelines on the components of CPD and attaining CPD points (World Physiotherapy 2021).

As physiotherapy includes hands-on practice, most teaching and learning opportunities such as BT and manual modality practice prior to the COVID-19 pandemic relied on physical human contact, however this was not possible during the pandemic because of the social distancing rules. The evolving situation called for adaptations to continue CPD in a context where there was pre-existing ambiguity (Gopaul, Manie & Amosun 2020). In addition, the physiotherapists practising during COVID-19 had unique insight into the changes that were made to their CPD and whether they found them beneficial or not.

Therefore, the aim of our study was to examine the availability and perceived quality of CPD for physiotherapists who worked before and during the COVID-19 pandemic in Lagos, Nigeria. The components of CPD that will be focused on in our study are workplace teaching and learning, reading the professional literature, conferences, training, physiotherapy and inter-professional meetings. Potential applications of our study may be to guide CPD policies in Lagos, Nigeria and may therefore improve clinical outcomes.

## Research methods and design

A qualitative methodology was chosen for our study, utilising the paradigm of interpretivism as the ontology is based on people’s reality being subjective with multiple interpretations. This theory encouraged the authors to be aware of and to understand the different experiences of the participants, to gather diverse interpretations (Bunniss & Kelly 2010). Semi-structured interviews were used because of the different time periods being examined, as stated in the aim. The flexibility of this interview format was important so that all the questions asked stayed within a thematic framework, but could be asked out of order. This enabled the interviewees to give more information rich answers as they were able to talk about their experiences in a timeline that was most natural to them. Our study was granted ethical approval by the Leeds Institute of Health Science Research Ethics Sub-Committee at the University of Leeds.

### Sampling

Physiotherapists were chosen as participants for our study as the aim was to examine their experiences. Participants were conveniently sampled over a 5-week period by sending general participation request emails to the enquiry email addresses of hospitals, clinics, and rehabilitation societies in Lagos. The use of convenience sampling was justified because of the use of mass emails and the sample population being homogenous (living within a certain area and in the same profession) (Bujang et al. 2012; Jager, Putnick & Bornstein 2017). The participation request emails sent to organisations contained a summary of our study and the link to a secure Google Form for participants to complete basic information, which determined their eligibility. Some of these organisations then disseminated the information in the email to their employees and members, which enabled potential participants to access the information and the form. These organisations were found through internet searches and word of mouth from colleagues who live in Lagos. The decision was made to focus on Lagos State since the size of Nigeria means there are large discrepancies in services nationwide and Lagos has the largest population of people and physiotherapists (Odumodu et al. 2019).

If participants who completed the Google Form met the inclusion criteria (shown in Box 1**)** they were contacted via email to request interview availability and were sent the consent form. The criteria were decided with our study aim and Lagos physiotherapist factors in mind (Odumodu et al. 2019). Furthermore, participants were recruited through snowballing via initial participants (some participants who had been interviewed shared their experience and initial request email with colleagues, who then completed the Google Form) (Bowling 2014). Initially, the target number of participants was 20, this number was chosen because of the high information power of each participant and the limitation of there being one available interviewer (Malterud, Siersma & Guassora 2016). The final number was reduced to 10 as at this point in the data collection, there were no new emerging themes or sub-themes (Bowling 2014; Malterud et al. 2016).

The interviews were conducted online on Zoom. This platform was chosen over others because of its widespread use among the sample population and simple functionality. In addition, the topic of the interviews was not particularly sensitive, so the video-conference interviews approach enabled participants to feel comfortable and build rapport with the interviewers (Nehls et al. 2015).

BOX 1 Inclusion criteria.

**Table:** 

Currently practising physiotherapy
Fully qualified as a physiotherapist by 2016
Has been working in Lagos State as a physiotherapist for at least 2 years pre-COVID-19

*Source*: Odumodu, I.J., Olufunlayo, T.F., Ogunnowo, B.E. & Kalu, M.E., 2019, ‘Satisfaction with services among attendees of physiotherapy outpatient clinics in tertiary hospitals in Lagos State’, *Journal of Patient Experience* 7(4):468–478. https://doi.org/10.1177/2374373519847370

COVID, coronavirus disease 2019.

These criteria allowed a wide range of participants’ experiences that fitted the timeline specified in our aim.

### Data collection

Eleven interviews (including one pilot interview) were carried out via Zoom, using the video function. Table 1 displays the participant details. Each interview lasted about 20 min, and all interviews were carried out by the first author between May 2021 and June 2021. The video calls were audio and video recorded (with consent) and saved on a secure drive. There was no interpreter required and all interviews took place in English. Each participant gave informed consent by electronically signing a form before the interview date and additional verbal consent at the start of each interview, when the consent form was read aloud by the first author.

**TABLE 1:** Participant Details (*n* = 10).

Participant characteristics	Number
Male participants	4
Female participants	6
Junior-level physiotherapy participants	4
Mid-level physiotherapy participants	4
Senior-level physiotherapy participants	2
Participants working in public sector	4
Participants working in private sector	6

Note: All physiotherapists involved in our study worked before and during COVID-19.

The interview questionnaire (Appendix 1) was developed based on the interpretivist paradigm, our study aim, and experiential knowledge of the authors (Bunniss & Kelly 2010). The broad focus of the questions were the experiences of physiotherapists’ learning opportunities for CPD pre- and during COVID-19, separated into opportunities within or provided by the workplace and non-workplace-based opportunities. These were seen as themes-questions to gather information were included under each theme. Each theme was then colour coded to make data analysis more efficient.

The pilot interview was conducted at the beginning of the data collection process to test the questionnaire (Bowling 2014). As a result, some additional open-ended questions were included at the start of the questionnaire to improve rapport and changes were made to the order of questions. The pilot interview findings were not included in the data analysis to uphold validity.

### Data analysis

Qualitative thematic analysis was carried out using a mixture of inductive and deductive coding. This ensured all themes encountered could be adequately explored to give a thorough overview of the participants’ experiences (Fereday & Muir-Cochrane 2006). Every interview was transcribed from the recordings by uploading the voice recording to the online transcription platform Otter.ai by the first author. After the transcribed text was downloaded onto a word document (1 per interview) the authors checked the transcription multiple times while watching the video and made notes on additional features such as body language to ensure confirmability. After transcripts were checked, audio files that had been downloaded onto a secure laptop were deleted.

Subsequently analysis was undertaken, without the use of any analysis software. This data analysis method supports our aim as it reports the key elements of the participant’s experiences while allowing for new emerging themes to be noticed (Bowling 2014). During the analysis process, the transcripts were colour-coded according to themes used in the interview questionnaire and each theme was given an *a priori* code using deductive coding. Furthermore, emerging codes were assigned to new themes (within the colour coded scripts) that were derived directly from reviewing the transcripts, using inductive coding.

The transcripts were then re-organised according to the codes with group relevant information together under each defined theme: a document was made for each code, and all the transcribed text from all interviews that fell within the code was pasted into the document. This meant there was one document for each theme, and they could be analysed separately. If one piece of text was relevant to more than one code, it would go into the document for each of the codes (Bowling 2014).

## Results

This section is divided into three main sub-sections based on the initial themes used for the interview questionnaire: pre-COVID access to CPD through the workplace, pre-COVID access to CPD through non-workplace organisations, and the impact of COVID-19 on the two above themes. Table 2 displays the themes that will be discussed within the findings.

**TABLE 2:** Themes discussed in findings.

Theme	Sub-Themes
Theme 1: Pre-COVID-19 access to CPD through the workplace	Sub-theme 1.1: Type of CPD received in the workplace. Sub-theme 1.2: Difference in workplace CPD between public versus private sector
Theme 2: Pre-COVID-19 access to CPD via non-workplace organisations	Sub-theme 2.1: Non-workplace CPD Sub-theme 2.2: Barriers to attending non-workplace CPD
Theme 3: Impact of COVID-19 on CPD opportunities	Sub-theme 3.1: Workplace CPD Sub-theme 3.2: Non-workplace CPD
Theme 4: Participant recommendations	-

CPD, continuous professional development.

### Theme 1: Pre-COVID access to continuous professional development through the workplace

Availability of teaching for physiotherapists pre-COVID are presented under sub-headings.

#### Sub-theme 1.1: Type of CPD received in the workplace

Three types of teaching were mentioned by participants: BT, case reviews (CR), and presentations:

- Bedside teaching was the most common type of teaching experienced by participants, and the type of teaching preferred by most. A participant working in a public secondary hospital highlighted the reason for this:

‘The best way to translate what you’ve learnt is practically, which is through hands on bedside teaching.’ (Participant 3, mid-level physical therapist, public secondary hospital)

Participants who received BT often had their teaching carried out informally and inconsistently. This was found to be the case because BT was mostly dependent on the availability and willingness of more senior physiotherapists, as there were no specific teaching guidelines. It was also highlighted that with BT being based on patients who were admitted, a wide range of conditions was often seen, and more specialist equipment was used, which broadened knowledge and skills:

- Case reviews were the second most common teaching participants received, and often took place alongside BT or presentations. As with BT, junior physiotherapists relied on the availability of senior physiotherapists to present CR, resulting in more inconsistent teaching opportunities. However, it was found to be the most collaborative teaching style because of the sharing of knowledge that took place.
- The least common teaching style for participants was through formal presentations. This was found to be the most structured method because they had to be timetabled in advance weekly or monthly, and were often combined with CR:

‘At these monthly team presentations, a member of staff would present on upcoming trends with certain conditions. It was a great way to learn from the seniors.’ (Participant 4, mid-level physical therapist, public secondary hospital)

In terms of the frequency of organised (by their employer) teaching junior physiotherapists received, all participants felt that the frequency of their teaching was not high enough. There was no noticeable frequency pattern. Most participants received teaching at least once a month; some had weekly sessions, some every few days, and some not at all. Most participants did not have a set schedule of teaching, so frequency varied monthly:

‘I wasn’t really supervised except on my first day. I’m pretty much on my own when it comes to teaching.’ (Participant 1, junior physical therapist, private rehabilitation clinic)

#### Sub-theme 1.2: Difference in workplace CPD between public versus private sector

There were many discrepancies in the availability and quality of teaching participants received, depending on whether they worked in the public or private sector. More often, private organisations did not provide structured or frequent teaching. A reason given for this was that physiotherapists working privately often went to see their clients at home as opposed to a hospital setting, which removed the opportunity for BT. This affected participants’ confidence for more complex conditions. A participant explained this by saying:

‘We often go to see our clients at home so have a smaller pool of people to receive teaching from. We work very independently and do not have access to as much supervision as we would like.’ (Participant 2, mid-level physical therapist, private rehabilitation clinic)

However, for participants working privately who did receive teaching, the mix of styles was like those working publicly.

Notably, two participants who worked at public specialist orthopaedic hospitals had positive teaching experiences. It was emphasised that they received teaching most days at the hospital (formal and informal) because there were many experienced senior staff, which created a vigorous learning environment:

‘There is very structured teaching. It is hands on and there are a lot of staff to provide it. We have BT on ward rounds every morning and additional teaching throughout the day.’ (Participant 5, junior physical therapist, public orthopaedic hospital)

‘I have noticed that specialist hospitals give better support because the staff knows what it’s like to be a young physiotherapist.’ (Participant 6, junior physical therapist, public orthopaedic hospital)

### Theme 2: Pre-COVID-19 access to CPD via non-workplace organisations

This theme is presented under sub-headings, which were identified as emerging themes during transcript analysis. The structured events referred to under this theme include any of the following: conferences, courses, webinars, seminars, journal club activities.

#### Sub-theme 2.1: Non-workplace CPD

In terms of resources, no participant was given access to online physiotherapy databases or an in-person library by their employer or through being a member of a physiotherapy and/or rehabilitation society. Only participants who paid individually had access to these resources. This finding was the same in participants who worked publicly and privately:

‘There was no access to anything like a library or online database. You had to search out information you needed by yourself.’ (Participant 7, junior physical therapist, public secondary hospital)

There were national conferences (which were seen as priced affordably) available to all the participants and most of them attended as many as they could. Numerous incentives were given as reasons for attending conferences and events. The main incentive that was mentioned by most participants was CPD points. These are points awarded by the MRTB that participants needed a minimum of, to renew their physiotherapy licences. Because of this, it was mentioned that certificates being given was an incentive as they were proof of attendance:

‘You go for CPDs every once in a while. And we’re told to attend because we need the points from the events and there are monetary penalties attached to not reaching the MRTB threshold.’ (Participant 3, mid-level physical therapist, public secondary hospital)

#### Sub-theme 2.2: Barriers to attending non-workplace CPD

Most participants attended structured learning events, but there were barriers preventing frequent attendance principally, a lack of availability and high costs.

Regarding availability, there are not many physiotherapists in Lagos; however, some participants did not attend events because of there not being enough spaces. It was highlighted that the conferences organised by the National Society of Physiotherapy (NSP) were not large enough to accommodate more than 60–80 people, and one event took place every 3–4 months:

‘No, no. There are not enough spaces for these events, nor do they happen frequently enough.’ (Participant 9, Senior physical therapist, public secondary hospital)

A few participants also noticed that the topics covered at events were basic skills and/or common conditions. Therefore, mid and senior level physiotherapists did not feel they gained much knowledge. For that reason, they preferred attending international conferences:

‘We keep having conferences that are just above entry level. I want to see more conferences that go in depth about certain conditions.’ (Participant 2, mid-level physical therapist, private rehabilitation clinic)

On the other hand, some participants did not attend conferences and learning events because they could not afford to pay for the tickets or transportation required. A few participants were able to travel for conferences and they were senior level physiotherapists:

‘I was able to go for quite a few of them. But sadly, it was self-sponsored, and it cost quite a fortune.’ (Participant 2, mid-level physical therapist, private rehabilitation clinic)

### Theme 3: Impact of COVID-19 on CPD opportunities

In March 2020, when the COVID-19 pandemic reached Nigeria, the country went into lockdown for 2 months where only essential workers left the house and most public places (restaurants, parks) were closed. For the remainder of 2020 and beginning of 2021, social distancing protocols were maintained in healthcare facilities.

The findings in this theme are divided into two broad sections based on the initial deducted themes used in the interview questionnaire: workplace learning and non-workplace learning.

#### Sub-theme 3.1: Workplace CPD

In terms of availability and quality of teaching during COVID-19, there was a wide variety of experiences. Participants who worked in specialist hospitals overall saw no difference in the quality of their teaching, except for initially when patients missed appointments because of health fears. Likewise, other participants found teaching halted initially, then resumed, but to varying degrees. Social distancing restrictions meant that there could only be a certain number of people in any given room, so patients presented physically less often, therefore BT reduced drastically:

‘The focus was on decongestion and then people would only come to work when a few people would be physically present, so we did a lot less teaching and learning.’ (Participant 4, mid-level physical therapist, public secondary hospital)

In contrast, there were some participants working publicly and some working privately who saw no change in the availability and quality of their teaching. For those working privately, it was because prior to COVID-19 they were already learning independently. For those working publicly, a few participants found there was no impact on their teaching as their primary teaching style was not bedside and could be carried out socially distanced while wearing masks. While some found that even BT continued as normal with everyone wearing masks:

‘I have noticed every hospital and clinic has its own approach … Honestly teaching in my unit has remained unchanged.’ (Participant 7, junior physical therapist, public secondary hospital)

Because of social distancing, the bulk of teaching for most participants moved online. As a result, some participants found that the frequency of training they received increased because of the ease of presenting cases and sharing information online:

‘For the first time every member of the team is able to attend the meetings and be taught.’ (Participant 10, senior physical therapist, working publicly and privately)

One participant expressed that receiving teaching online made learning more inclusive and collaborative:

‘We have branches in Lagos and Abuja and for the first time every member of both teams is able to be in the same meetings and be taught at the same time.’ (Participant 8, mid-level physical therapist, public secondary hospital)

However, participants reported two main limitations to online teaching. Firstly, there was more independence and isolation for teaching because not every online session was compulsory, whereas when they received teaching in person it was. It was explained that the supervisors could not make teaching online compulsory because people did not always have access to the Internet or a computer if they were working from home.

Secondly, there was a lack of physicality, which was expressed in more junior physiotherapists who were more recent graduates and felt they were missing out on the benefits of practising what they were learning on live people:

‘I think nothing beats hands on training, especially for physiotherapy. You can do as much as you want online but if I can’t translate it into treating a physical patient because I do not know how to, then it’s almost like I haven’t learnt anything.’ (Participant 7, Junior physical therapist, public secondary hospital)

#### Sub-theme 3.2: Non-workplace learning opportunities

From March 2020, every in-person event was cancelled with a few moving online. Consequently, many participants attended international conferences and seminars online that they may not have been able to attend previously because of the high cost involved:

‘Now, because of COVID we can join international conferences and there are even opportunities to make presentations. You no longer must travel all the way.’ (Participant 4, mid-level physical therapist, public secondary hospital)

Most participants found online learning events productive and more accessible than physical events. It was mentioned numerous times by participants that online events were easy and increased their awareness of physiotherapy knowledge and opportunities for online learning:

‘COVID played a big part in making me more aware of online opportunities, both online libraries and online conferences.’ (Participant 3, mid-level physical therapist, public secondary hospital)

Additionally, some incentives mentioned by participants who attended online events was the lack of expenses and ease of attendance:

‘COVID has made it almost normal to not have to travel and spend a lot of money on learning opportunities.’ (Participant 2, mid-level physical therapist, private rehabilitation clinic)

‘I was able to hook up to multiple conferences on the same day because I did not have to travel from location A to B.’ (Participant 11, mid-level physical therapist, private rehabilitation clinic)

As there were not many online learning opportunities from Nigeria, most participants joined international conferences. However, some participants were not aware of online opportunities and found it difficult to reach the required number of CPD points. In addition, the turnout for Nigerian online events was not high because the required technology and a lack of network stability required to join for events that lasted longer than an hour acted as a disincentive:

‘Many of us were demoralised by COVID. The willingness to join was not there. Also, many people in physiotherapy are not tech savvy.’ (Participant 9, senior physical therapist, public secondary hospital)

### Theme 4: Participant recommendations

Participants were asked to give recommendations they felt would improve access and quality of their continued professional development.

A few physiotherapists brought up the concept of a national teaching guideline to provide support for staff:

‘There are so few guidelines on teaching that creates a disincentive; only those that are confident teach and I would like organisations to focus on good quality teaching in both urban and rural areas.’ (Participant 5, junior physical therapist, public orthopaedic hospital)

Similarly, some participants recommended the implementation of a formal national teaching programme software that combined E-learning with physical learning.

Furthermore, a few participants suggested an increase in advocacy and promotion by hospitals and physiotherapy societies to provide a wider range of events themselves:

‘I also think hospitals and associations need to find out the gaps and organise things in those areas.’ (Participant 10, senior physical therapist, working publicly and privately)

## Discussion

The aim of our study was to examine the availability and perceived quality of continuous professional development for physiotherapists who worked before and during the pandemic in Lagos, Nigeria.

The main barriers for physiotherapists attending non-workplace learning events such as conferences pre-COVID-19 were identified. These barriers were unsurprising and are supported by studies carried out in Africa. A study with 143 physiotherapists in Southwest Nigeria in 2017 found the main barriers to be financial constraints (37%) and a lack of events in an area of interest (22%). This study also found that 94% of physiotherapists attended fewer than five additional learning events a year, which they perceived affected their confidence level and quality of rehabilitation provided (Akodu, Ileyemi & Ekanem 2017). A different study from Ghana found that when leaders in the sector and organisations supported structured learning events practically and financially, it overcame these barriers, improving access and attitudes towards continuous learning (Bello & Lawson 2013).

Bedside teaching was the most popular workplace teaching style received by physiotherapists in our study. This is the same in most African countries (Amosun 1994). A general lack of frequency and structure for teaching was also highlighted, which had a lot to do with a lack of guidelines and support. Our findings are supported by a study carried out in Uganda in 2020 that showed that physiotherapy clinical educators found it difficult to attain high teaching standards because of a lack of guidelines nationally and from their organisations (Kibuuka 2020). In South Africa, 25 physiotherapists participated in focus groups to formalise clinical practice guidelines. A similar approach could be taken in Nigeria: including physiotherapists in policy and decision-making to standardise teaching nationally (Van Aswegen et al. 2017).

With the emergence of COVID-19 in 2020, most teaching and learning opportunities moved online, and we found that physiotherapy in Lagos, Nigeria, was no different. The main challenge identified, particularly by junior physiotherapists, is properly translating the physicality of physiotherapy into online teaching so they feel adequately equipped when seeing patients. These limitations were addressed by a study carried out in Brazil, Cyprus, and the United States. Plummer et al. (2021) used innovative teaching Apps and incorporated participant feedback to improve their students’ experiences. These and other context-specific measures could be used to further enhance online teaching for physiotherapists in Nigeria, as the benefits (increased availability and reduced costs) are worthwhile continuing post-COVID-19.

Moving structured learning opportunities such as conferences online to overcome barriers to access such as high monetary cost and transportation fees was a major finding of our study and is an argument that was made by studies caried out pre-COVID-19 (Maharaj 2013; Kenyon 2011). These studies advocate for an increased integration of E-learning into all forms of teaching to ensure equal access to learning events. The effect would be to provide all the staff the opportunity to improve their technological skills, which addresses the concerns raised by some participants surrounding the lack of skills and inertia to moving events online.

Our study was limited by a small sample size even though we achieved data saturation as well as the inclusion of participants from hospitals, clinics, and rehabilitation societies with email addresses only. This might have created selection bias as some areas of Lagos could have a larger proportion of physiotherapists without email addresses, causing the sample population gathered to be less representative of the state. A study with more time and human resources may be able to collect more representative data and therefore have more generalisable data.

A future study exploring the opinions and experiences of regulators and physiotherapists who provide teaching and training would increase the scope of our study and provide more perspective for those who make CPD policies for physiotherapists in Nigeria.

## Conclusion

Our study identified that pre-COVID-19 there was no set standard for how physiotherapists received teaching and learning opportunities as the national guidelines for CPD only provide guidance on the number of CPD points needed for each physiotherapist to maintain their licence. However, the most taught and most preferred teaching style was BT. After the outbreak of COVID-19 in March 2020, continuous learning partially adapted to move online. The main benefits identified include increased availability and lowered costs and the main drawback was a lack of practicality.

Changes are needed to improve the quality and availability of continuous professional development for physiotherapists in Lagos, Nigeria. Therefore, a blended (online and in person) teaching style combined with organisational advocacy for a national teaching guideline is recommended. The software used to deliver online teaching could additionally be used as a database for physiotherapy textbooks and research articles. This would improve collaboration between physiotherapists at all levels and close the gap between the continuous learning received in different sectors, and may improve patient outcomes in the long run.

## Appendix 1: Questionnaire

### Color Code

Opening – black

Theme 1 – availability and quality of training pre COVID

Theme 2 – opportunities for further learning pre COVID

Theme 3 – availability of training post-COVID

Theme 4 – opportunities for further learning post COVID

Theme 5 – gaps they have identified in training/learning opportunities and recommendations

### Opening

Introduce the interview:

1. Give your name and ask for their name.
2. Explain the aims of interview.
3. (Review the information sheet and consent form and ensure that consent is provided)
4. Remind the respondent that they can stop at any time.
5. Ask if the respondent has any questions.

### Sample participant

1. **Can you tell me what process you had to go through to become a physiotherapist?**
2. **Do you wish you had trained elsewhere and why?**
3. **Public or Private?**

1. **Was there hand-on training during the course**
2. **Was there hands-on training after qualification**
3. **What type of teaching**
4. **How often was the teaching**
5. **Who provided this teaching**
6. **Was it structured/formal**

1. **Did COVID affect availability of teaching, if yes how?**
2. **Did COVID affect quality of teaching (frequency, who provided, structured/formal), if yes how?**

1. **In addition to professional teaching were there any individual opportunities for learning such as courses, conferences, seminars etc**
2. **Certificates or any incentives given to go for them?**
3. **General opinion of them/if they were found useful**

1. **In addition to professional teaching were there any individual opportunities for learning such as courses, conferences, seminars etc**
2. **Certificates or any incentives given to go for them?**
3. **General opinion of them/if they were found useful**
4. **Their opinion of access post-COVID and how/why**

1. **Would you like more teaching/learning opportunities?**
2. **In what areas and through what mediums?**
